# TopoToolbox: Using Sensor Topography to Calculate Psychologically Meaningful Measures from Event-Related EEG/MEG

**DOI:** 10.1155/2011/674605

**Published:** 2011-04-18

**Authors:** Xing Tian, David Poeppel, David E. Huber

**Affiliations:** ^1^Department of Psychology, New York University, 6 Washington Place Suite 275, New York, NY 10003, USA; ^2^Department of Psychology, University of California, San Diego, La Jolla, CA 92093, USA

## Abstract

The open-source toolbox “TopoToolbox” is a suite of functions that use sensor topography to calculate psychologically meaningful measures (similarity, magnitude, and timing) from multisensor event-related EEG and MEG data. Using a GUI and data visualization, TopoToolbox can be used to calculate and test the topographic similarity between different conditions (Tian and Huber, 2008). This topographic similarity indicates whether different conditions involve a different distribution of underlying neural sources. Furthermore, this similarity calculation can be applied at different time points to discover when a response pattern emerges (Tian and Poeppel, 2010). Because the topographic patterns are obtained separately for each individual, these patterns are used to produce reliable measures of response magnitude that can be compared across individuals using conventional statistics (Davelaar et al. Submitted and Huber et al., 2008). TopoToolbox can be freely downloaded. It runs under MATLAB (The MathWorks, Inc.) and supports user-defined data structure as well as standard EEG/MEG data import using EEGLAB (Delorme and Makeig, 2004).

## 1. Introduction

This tutorial introduces a free open-source toolbox that includes functions for topographic analyses of event-related electrophysiological data (EEG/MEG). These analyses do not anatomically locate neural sources. Instead, by providing robust measures of response similarity between conditions and response magnitude for each condition, multivariate analyses are used to test psychological theories. These techniques are not new and were previously proposed and validated [1, 2]. However, their implementation within a user friendly toolbox is new. The core routines of TopoToolbox calculate a measure of angle between EEG/MEG topographies in *n*-dimensional sensor space, where *n* is the number of sensors. This toolbox is called TopoToolbox and it uses MATLAB (The MathWorks, Inc.) to analyze either user-defined or EEGLAB [3] standardized data sets. It can be downloaded from https://files.nyu.edu/xt235/public/ where a detailed tutorial, manual, and example data can be found. 

Multivariate methods are frequently used to analyze fMRI experiments [4–8], and similar multivariate methods are beginning to appear in EEG/MEG studies. However, unlike fMRI studies in which multivariate analyses involve multiple anatomically defined voxels, multivariate analyses in EEG/MEG involve multiple sensors (e.g., electrodes or SQUID magnetometers) that each reflect a mixture of underlying neural sources. Thus, for EEG/MEG, these analyses are often in sensor space rather than source space. 

We briefly review several previously proposed EEG/MEG multivariate analysis methods. Several of these are closely related to the analyses contained in TopoToolbox, and we further consider these relations in the Discussion. Global field power (GFP; [9]) was one of the first measures to use multiple sensors in EEG data. GFP is the standard deviation of all sensors from the global mean. Lehmann and Skrandies [9] also proposed a topographic measure termed global dissimilarity (DISS), which is the square root of the mean of the squared differences between the sensors after first scaling the sensor values in each condition by dividing by the GFP of that condition (i.e., Euclidean distance between the two sensor vectors after normalizing them to have length 1.0). A nonparametric method called TANOVA (topographic ANOVA) has been proposed to statistically test the significance of the DISS value between two grand average topographies by calculating a null hypothesis distribution from repeated random permutations of the data [10–12]. Similar to the analyses contained in TopoToolbox, DISS is a measure based on the sensor space. In contrast, some recent multivariate analyses have been developed that transform the data of multi-sensor event-related EEG/MEG experiments using a basis set, such as with independent components analysis (e.g., [13] for a review see [14]). Thus, these techniques operate in component space, where each component is a derived topographic pattern, rather than performing tests based on the raw topographic sensor space.

Compared with traditional waveform-based analyses, topographic analyses have the following advantages. First, topographic analyses use all of the data in a single test and do not suffer from problems related to “double dipping” that can occur with multiple comparisons [15, 16]. Second, EEG waveform analyses are highly dependent on reference channel selection (see the review by Murray et al. [17]) and MEG waveform analyses are difficult to combine across individuals in sensor space due to large differences in the response of the same sensor for different individuals [18]. Third, waveform analyses cannot determine whether a change between conditions is more likely due to a change in neural response magnitude or a change in the distribution of underlying neural sources that gave rise to the response [1]. Even if the goal is anatomical localization, analyses based on sensor topography can provide an important intermediate step and validity check prior to source analyses. Furthermore, multivariate analyses can be used to test psychological theories (e.g., “how”) in the absence of anatomical localization (e.g., “where”). 

There are a range of techniques that use multiple sensors to anatomically locate neural responses [19]. However, these techniques often make strong assumptions such as temporal and anatomical independence between the underlying neural sources. Working within a component space based on sensor topography, rather than source space, independent components analysis [20] has proven useful for extracting independent noise components such as the beating heart or eye blinks [21]. Pascual-Marqui and colleagues [22] proposed a data-driven, hypothesis-free topographic analysis of electrophysiology that blindly separates the grand average into different response components by using multiple spatial templates as applied to each individual data set.

Aside from the choice of analyzing a select few sensors versus the entire multivariate sensor topography, another choice in electrophysiological experiments is whether to analyze each individual separately versus the entire data set across all participants. Because individuals differ both in terms of anatomical structure and in terms of task related neural responses [23, 24], averaging across individuals can produce unreliable results, particularly with MEG data (see [1] for a reliability comparison between a sensor selection analysis and the projection test contained in the TopoToolbox). However, if the goal is to infer something about the adult population in general, then it is necessary to use a statistical test with subject as a random factor. If spatial and temporal individual differences are not considered when averaging across individuals, at best this will reduce the signal-to-noise ratio and at worst it might bias the results. The aforementioned multivariate methods do not provide measures that can be compared across individuals in a reliable manner in light of these individual differences. One of the primary advantages of the TopoToolbox is its ability to normalize against individual differences and derive a single magnitude measure that is psychologically meaningful [1]. To date, this method has been successfully applied to MEG data across a variety of experimental paradigms [1, 2, 25, 26], demonstrating its ability to produce reliable measures that can be compared across individuals. 

## 2. Methods

This section describes the equations and algorithms implemented in the TopoToolbox. Some details are omitted, such as navigation of the menus in the toolbox and particular parameter selections. Descriptions of these details and example data can be downloaded from https://files.nyu.edu/xt235/public/. The core of the toolbox is a two-stage analysis that first quantifies the topographic similarity and second quantifies response magnitude through topographic projection. In this tutorial, we also describe a new addition to the toolbox that assesses dynamic variations in the observed topography. 

For the first stage (*angle test*), similarity measures are calculated between the results of different conditions. Significant dissimilarity indicates that the observed pattern across the sensors qualitatively changed, such as what might occur with differing mixtures of underlying neural sources. For instance, if one condition evokes a response in auditory cortex while another condition evokes a response in visual cortex, then this analysis would conclude that the patterns were dissimilar even when measured at the same latency. However, if the patterns are not found to be dissimilar, then the second stage calculates geometric projections between patterns, which are used to indicate whether there has been a change in response magnitude (i.e., more or less of the pattern). This is done separately for each individual based on that individual's “template” response. Because these projections are normalized for each individual, the conclusion of this second stage is a statistical test across individuals.

Because these methods use traditional null hypothesis testing (in future work, we plan to supplement the TopoToolbox with Bayesian statistics), a failure to find a significant difference with the angle test does not necessarily indicate that the conditions of interest did not differ (i.e., there is an unknown type II error rate). Furthermore, if exceedingly unlucky, two different distributions of neural sources can in theory produce exactly the same topographic pattern (e.g., an inverse problem). Putting aside this remote possibility, the two tests can be used in combination to determine whether the best interpretation of a change between conditions is a change in the distribution of neural sources versus a change in response magnitude. More specifically, because both tests operate on the same data, they have equivalent statistical power, and a result in which the angle test fails to find a difference but the projection test produces a significant difference supports the conclusion that there was a change in magnitude.

### 2.1. Angle Test: Topographic Similarity

**Figure 1:**
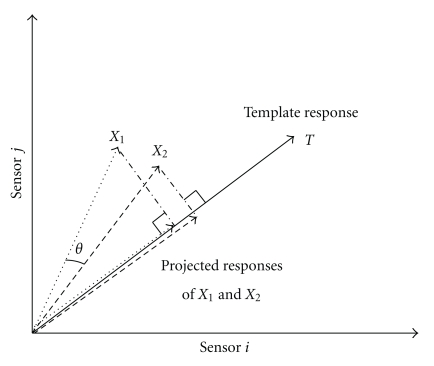
Illustration of *angle test* and *projection test *with 2 sensors, although the technique is typically applied to an n-dimensional sensor space where n is the number of sensors. Each experimental condition (*X*_1_ and *X*_2_) produces a magnitude of response for each sensor. The angle (*θ*) between these conditions is used as a measure of pattern similarity. Some other condition defines a template pattern (*T*), and projections onto this template provide numbers for “how much” of the template each condition produced. These response magnitudes can then be compared across individuals.

The topographic analyses in TopoToolbox assume that each of the *n* sensors provides a unique dimension of variation. Thus, the 2D or 3D spatial arrangement of the sensors is irrelevant. Instead, all sensors are equally important regardless of their position. The *n*-dimensional spatial patterns across sensors for different experimental conditions (e.g., the pattern for condition *X*_1_ versus the pattern for condition *X*_2_) are first assessed with an *angle test*. The *n*-dimensional sensor space angle (*θ*) is calculated to measure similarity between these patterns (see Figure 1 for an example with 2 sensors, which is the largest number of sensors that can be accurately portrayed on the written page). If the two conditions produce a similar distribution of neural sources, then the angle in sensor space will be 0 degrees even if one condition produces a larger response magnitude than the other condition. However, if the two patterns are completely opposite (i.e., sign flip), then the angle is *π*. The angle is measured by calculating the cosine of the angle, which is a normalized dot product between the two sensor vectors (1). If the sensor data are zero centered (e.g., using an average reference channel), this is formally the same as the Pearson correlation coefficient. We term this cosine angle the *angle measure*. The *angle measure* ranges from −1 to 1, where −1 is observed for completely opposite patterns and 1 is observed for the perfectly similar patterns (regardless of magnitude). Because this *angle measure* is calculated between conditions, we term it the *between* angle measure:

(1) $$\cos\;\;\theta =\frac{{\vec{X_{1}}}^{T}\vec{X_{2}}}{\left|\vec{X_{1}}\right|\left|\vec{X_{2}}\right|}.$$

A null hypothesis is needed to statistically assess the *between* angle measure (i.e., is the angle between conditions greater than expected based on chance). There may be other methods for constructing a null hypothesis, but a simple solution is to separate the experiment into two halves and then calculate *between* versus *within* angle measures based on the average patterns found for each half, condition, and individual. The *angle measure* is calculated separately at each evoked time point, and then these separate *angle measures* are averaged over a temporal window to increase reliability. Both the beginning and the end point of the averaging window can be set. In particular, the middle of the averaging window can be adjusted separately for each individual considering that different people tend to produce waveforms that achieve peak values at different times (see [1] for evidence of individual differences in the duration to reach a peak response). If the separation of the experiment into two halves is done according to trial number (first versus second half of the experimental session), this produces a null hypothesis that includes variance due to changes over time, such as what might occur with head position shifts. However, the toolbox also allows that the separation into halves can be done in an interleaved manner (odd number trials versus even number trials) or through a random split of trials. The null hypothesis is based on the *within* angle measure that compares the responses between the two halves for the same condition for each individual whereas the *between* angle measure compares the responses between the two halves for different conditions for each individual.

To understand the nature of these calculations, consider a comparison between two conditions (*X*_1_ and *X*_2_) across the experimental halves (a and b) with 10 individuals in the experiment. The null hypothesis *within* angle measure for the first individual is found by averaging the *X*_1a_/*X*_1b_ angle measure with the *X*_2a_/*X*_2b_ angle measure, and the experimental *between* angle measure is found by averaging the *X*_1a_/*X*_2b_ angle measure with the *X*_2a_/*X*_1b_ angle measure. These same values are calculated for the other 9 individuals, and then differences between the 10 *within* and 10 *between* angle measures are statistically assessed. It is important to note that although a direct measure of angle would require a statistical test designed for circular data, the angle measure in TopoToolbox is the cosine angle, which is a noncircular interval scale for the null hypothesis of no difference. Furthermore, if the sensor data are zero-centered, then the angle measure is the same as the Pearson correlation coefficient, which is traditionally tested using a *t* distribution. Therefore, TopoToolbox uses a paired *t*-test to determine if the response patterns were significantly dissimilar across the population for the two experimental conditions.

If the *between* angle measure is significantly smaller than the *within* angle measure, then the two experimental conditions are significantly dissimilar, leading to an unambiguous conclusion that a different mix of neural sources was responsible for the change between conditions. Furthermore, such a result suggests that the projection test, described next, should not be run and would produce ambiguous results because it confounds response magnitude with response similarity. Alternatively, the failure to conclude that the two conditions are significantly dissimilar implies that (a) a similar distribution of neural sources produced the response pattern in both conditions (b) that two different distributions of neural sources happened to produce the same topographic pattern (a remote possibility), or (c) that the *t*-test was not sufficiently powerful to detect dissimilarity. The question of statistical power can be addressed with the projection test. More specifically, if the projection test concludes that the response magnitude is significantly different between the two conditions, this suggests that there was sufficient power to have detected a difference in response similarity.

### 2.2. The Projection Test: Normalizing against a Template to Measure Response Magnitude

Most event-related electrophysiological studies analyze response magnitude of a select few sensors in different conditions. For these analyses, it is tempting to conclude that increases in response magnitude (either greater positivity or greater negativity) correspond to increases in the underlying neural response. However, when considering just a few sensors, it is unclear whether an increase reflects an increase in the magnitude of the neural response or whether an increase might instead reflect a shift in the distribution of neural sources, with some new source producing the apparent increase. Simply put, the question is whether the brain did the same thing to a greater extent in one condition, or whether the brain did two different things in the two different conditions. The answer to this question can be used to distinguish between competing psychological theories. As described above, the *angle test *can be used to determine if the distribution of neural sources changed between conditions. If the conditions appear to be sufficiently similar (not significantly dissimilar), then *the projection test* can be used to determine if the magnitude of the underlying neural sources has increased or decreased.

Besides providing a conclusion based on neural response magnitude (upon failure of the *angle test*), another advantage of the *projection test* is its ability to normalize against individual differences, thus providing a more reliable measure. This is achieved by projecting (2) the sensor pattern in each condition (*X*_*i*_) onto a template pattern (*T*) for that participant (Figure 1). As with the *angle measure*, this is done separately at each time point and window averaging is used to further increase reliability. The template is typically a response pattern across the sensors in some other condition using the same response window for averaging. Critical to the success of this projection is choice of the template. The template should include the same psychological processes as the conditions of interest (see an example below). Because a different template pattern is used for every individual, the projection values should lie on the same scale (i.e., more or less of that individual's template response). Therefore, individual topographic differences are eliminated through normalization. Furthermore, provided that the template is a “clean” pattern that is relatively devoid of overlapping waveform responses (in contrast to experimental conditions), the projection can serve to decontaminate response magnitude by eliminating overlapping waveform responses that are orthogonal to the template:

(2) $$\left|\vec{X_{i}}\right|\cos\;\;\theta =\frac{{\vec{T}}^{T}\vec{X_{i}}}{\left|\vec{T}\right|}.$$

Like the *angle test*, the *projection test* is statistically tested across individuals using a paired *t*-test. However, in the case of the *projection test*, the comparison is not a *between *angle measure versus a *within *angle measure but rather the projection value in one condition versus the projection value in the other condition for each individual.

An immediate priming experiment [26] provides an example of an appropriate template response and use of the angle and projection tests. In this experiment, every trial presented a prime word for 1,850 ms followed by the appearance of a second prime word for 150 ms (both prime words remained on the screen for the final 150 ms). Next, both primes disappeared, and a target word was briefly flashed and then masked. There were three conditions depending on whether this target word repeated the long duration prime word (the *long* condition), the short duration prime word (the *short* condition), or neither of the prime words (the *novel* condition). MEG was recorded in this experiment, and the measure of interest was the M170 to the target word. However, because the short duration prime appeared 150 ms prior to target word, and because a mask followed the target word, there was substantial contamination of the M170 pattern to the target word (due to the M400 to the short duration prime word and also the M100 to the mask). In contrast, the M170 to the first prime word provided a “clean” M170 that was used as a template pattern to normalize each individual's target word M170. Because there were individual differences in the timing of the M170, each individual was given a different 22 ms template time window according to that individual's peak M170 (as determined by the root mean square across all 157 sensors).

A priming effect is defined as the difference between a primed condition (e.g., the *long* condition) and an unprimed condition (e.g., the *novel* condition). Before concluding whether priming caused the M170 to decrease or increase, the angle test was used to assess whether each priming condition was dissimilar from the unprimed condition. These *within* and *between* angle measures were calculated for each individual using the same individually specified 22 ms time window as determined by that individual's template response (except that this window was placed in relation to the onset of the target word rather than the onset of the first prime word). Because the resultant angle tests failed to find any similarity differences, the projection test was used for each priming effect. As predicted by a neural habituation model of priming [27], these tests revealed that there was a significant neural response reduction in the target word's M170 when it repeated a long duration prime word but not when it repeated a short duration prime word [26]. Without these topographical analyses, this theoretical conclusion would not have been possible because (1) topographic differences made statistical test across individuals unreliable, (2) overlapping waveforms produced a target word M170 that was contaminated and thus unreliable, and (3) a statistical conclusion based on the magnitude of a few sensors might have confounded a change in the neural source distribution with a change in the neural response magnitude.

### 2.3. Angle Dynamics Test: Assessing Pattern Similarity over Time

For classic well-defined responses such as the M170 to a visual stimulus, the *angle test* and *projection test *can be used to measure similarity and response magnitude. However, in other circumstances, the waveform peaks are less well-defined and it can be difficult to determine when a certain response pattern reaches its peak and how long that pattern lasts. The dynamics of response patterns can be assessed by using the same *angle test* for similarity except that the test is applied at every time point rather than only at a well-defined peak. That is, the *angle measure* between a template and a condition of interest can be calculated at each sample time point for that condition (3). Just as with a well-defined peak, the *within* and *between* angle measures can be calculated at every time point to determine when the pattern defined by template is maximally exhibited in the condition of interest and during what time periods the template pattern does not exist:

(3) $$\cos\;\;\theta \left(t\right)=\frac{{\vec{T}}^{T}\vec{X_{i}}\left(t\right)}{\left|\vec{T}\right|\left|\vec{X_{i}}\left(t\right)\right|}.$$

A simple motor experiment [2] provides an example demonstrating the usefulness of this dynamic pattern analysis. This MEG experiment investigated the temporal characteristics of the neural sources involved in motor execution and imagery although only the motor execution results are summarized here. In this experiment, participants were asked to press a button at a comfortable pace upon hearing an auditory cue. They were encouraged to respond at a similar speed throughout the entire experiment. The MEG motor response was measured both by using an average that was time locked to the auditory cue (cue locked) and by using an average that was time locked to the button press (response-locked). The *angle dynamics test* was implemented by using the response-locked motor response as a template pattern (i.e., a classically defined motor response template) that was compared to every time point in the cue-locked epoch. An important validation of this angle dynamics test was whether it could be used to recover the same peak time in the cue-locked epoch as defined using classical methodology. The classically defined peak was identified using the root mean square (RMS) across the sensors to find a peak response. However, in the cue-locked epoch it is not always clear when to look for this peak, and so an RMS peak was chosen for each individual that was near the average reaction time of that individual. The important question was whether the angle dynamics test could find these RMS-defined motor response times in the cue-locked epoch without knowing the average reaction time of each individual.

**Figure 2:**
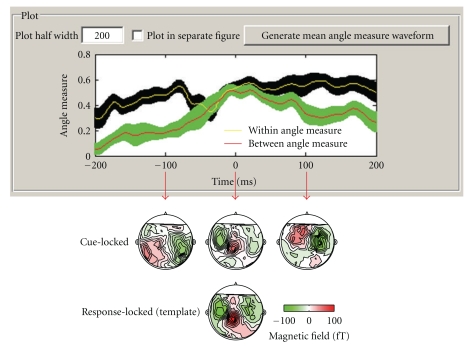
Results comparing the timing of motor response peaks as determined classically through the root mean square (RMS) across MEG sensors versus motor response peaks as determined by the *angle dynamics test*. The *angle dynamics test* used a template defined by the response-locked epoch, which was compared (*angle test*) at each time point along the cue-locked epoch. Because the cue-locked epoch has been adjusted according to each individual's RMS defined motor response peak, the zero point on the *x*-axis is the classically defined motor peak. Validating the *angle dynamics test*, the difference between the *within* and *between* angle measures becomes nonsignificant during a 101 ms window around the zero point. Shaded regions show the 95% confidence intervals for the *between* and *within* angle measures. The grand average of template and cue-locked responses at 3 different times are depicted on the bottom. As seen in these grand average responses, the cue-locked topography is similar to the response-locked template at the zero point but dissimilar 100 ms before and 100 ms after the zero point.

As seen in Figure 2, *between* and *within* angle measures were found at each sample point within the cue-locked epoch (using the response-locked template). This was done separately for each individual, and then these values were averaged and graphed with 95% confidence levels to produce the plots. The zero point of the *x*-axis is the time at which the motor response reached its peak value as classically defined by RMS. This was done separately for each individual, and time is shown relative to these individually determined peak times. As seen in Figure 2, the *between* angle measure approaches the *within* angle measure 50 ms before the RMS-defined peak latency (i.e., the zero point on the *x*-axis) and falls below the *within* angle measure 50 ms after the peak latency. Furthermore, beyond validating the timing of the peak time using the *angle dynamics test*, the *angle test* at the peak latency was not significantly dissimilar from the response-locked template, whereas they were significantly dissimilar 100 ms before and after the peak latency. The grand average topographies in Figure 2 further confirmed the results of the *angle dynamics test*: the cue-locked response at 0 ms shared the same distribution as the template, whereas the responses at −100 ms and 100 ms were apparently different from the template. This suggests that distribution of neural sources responsible for the motor response was different from the distribution of neural sources just before and just after the response. In contrast, during the peak time as defined by the *angle dynamics test* applied to the cue-locked epoch, the pattern across the sensors was similar to the template as defined by the response-locked epoch [2].

This is an important validation of the *angle dynamics test,* and it may prove to be of use in experiments where there is a need to find the timing of peaks that are not strictly locked to stimulus onset. For instance, consider an experiment that involves left key presses versus right key presses in a difficult task that produces many errors. Response-locked epochs could be used to define the template pattern for a left or a right key press, and then the *angle dynamics test* could be calculated for each of these templates to assess the online decision process as individuals gain more information favoring one response or the other (see [28] for a related method for assessing decision evidence accumulation in EEG data).

## 3. Discussion

There have been recent and exciting developments in the use of EEG and MEG analyses based on the topographic pattern across the entire sensor array [17]. Many of these techniques are highly complex and attempt to extract the responses of specific anatomically located neural sources. The techniques in TopoToolbox also use the topographic pattern across the entire sensor array to extract more information from MEG/EEG data. However, rather than attempting to measure particular neural sources, the goal of these analyses is to more simply ask whether the distribution of neural sources changed between conditions and, if not, whether that distribution was more or less active. The resultant techniques are relatively simple and can be used to ask functional questions such as how (same or different from the *angle test*), how much (*projection test*), and when (*angle dynamics test*). 

There are several analysis methods and associated software that are closely related to the TopoToolbox, such as TANOVA in LORETA [29, 30] and Cartool (http://brainmapping.unige.ch/cartool). Amongst the three core tests contained in the TopoToolbox, the *angle test* is the component most similar to the measures contained in these alternative software packages. In particular, although the equation for the angle measure is not identical to the equation for the DISS measure used in TANOVA, it has been proven that there is a linear relation between these two measures [31]. However, unlike application of DISS in TANOVA, the TopoToolbox calculates the angle measure separately for each participant and uses a statistical test with subject as a random factor whereas the statistical test of DISS in TANOVA tests for between-condition similarity differences in the topographies after averaging across subjects and uses nonparametric bootstrap sampling to test reliability across individuals. Beyond accounting for individual differences, another advantage of the *angle test* in TopoToolbox is that by splitting the experimental session into two halves, the null hypothesis distribution properly includes nuisance factors such as fatigue and head movements.

Beyond similarities between the angle measure of TopoToolbox and the DISS measure used in TANOVA, the TopoToolbox also contains *the projection test* and *angle dynamics test*, which are not found in other software packages. Thus, although there are other methods for assessing similarity of topographic patterns, only the TopoToolbox has a technique for determining whether the topographic pattern has increased or decreased in its response magnitude, as determined with a measure that decontaminates by using a clean template pattern, and also a technique for determining when the topographic pattern becomes most similar to a template pattern. The combination of the angle test and the projection test is particularly useful because in combination they can determine whether the best explanation for a change between conditions is that the topographic pattern changed (suggesting a different distribution of neural sources) or whether the topographic pattern magnitude changed (suggesting an increase or decrease in the neural response).

Due to the “inverse problem,” it is difficult, if not impossible, to infer underlying neural sources from scalp measurements; for any topographic pattern there are infinitely many combinations of neural sources that can give rise to exactly that pattern. It is for this reason that the techniques of the TopoToolbox do not attempt to identify the underlying neural sources. Instead, the goal of TopoToolbox is qualitative comparisons that can assess whether the distribution of underlying neural sources is likely to have changed, which would produce a different topographic pattern, and whether the distribution of underlying neural sources is likely to have increased or decreased, which would produce the same topographic pattern but a change in response magnitude for that pattern. However, there is still an inverse problem with these techniques; it is conceivable that the same topographic pattern is observed in two conditions (i.e., a failure to find dissimilar patterns with the *angle test*) even though the distributions of underlying neural sources are different. Nevertheless, the chance of this occurring would seem to be low considering that the conditions being compared are typically within the same task that involves the same cognitive processes. To partly address this question, Tian and Poeppel [2] compared the results from the *angle test* to the results of a source analysis, revealing that the source analysis suggested differently located sources when the *angle test* suggested that distribution of neural sources was different. However, source analysis also suffers from an inverse problem, and so the ideal method for validating this limitation, as well as limitations due to the use of null hypothesis testing, would be to use a “ground truth” comparison such as with intracranial EEG. 

In the absence of further validation of these techniques with a comparison to intracranial EEG, we have demonstrated that *projection test* reduces variability by normalizing against individual topographic differences, individual timing of peak response differences, and contamination from overlapping waveforms [1] and we have also demonstrated that the *angle dynamics test* can recover the timing of a motor response (as reported here and also by Tian and Poeppel [2]). Most importantly, a growing number of studies have found these techniques to be reliable and useful (e.g., [2, 25, 26]).

## 4. Conclusions

This paper introduced a new topographically based analysis toolbox for electrophysiological studies (EEG/MEG). We demonstrated that these within-participant analyses can normalize individual differences and derive psychologically meaningful metrics (similarity, magnitude, and timing) from high-density sensor arrays in a manner that overcomes several limitations of traditional waveform analyses.
